# AnNoBrainer, An Automated Annotation of Mouse Brain Images using Deep Learning

**DOI:** 10.1007/s12021-024-09679-1

**Published:** 2024-08-07

**Authors:** Roman Peter, Petr Hrobar, Josef Navratil, Martin Vagenknecht, Jindrich Soukup, Keiko Tsuji, Nestor X. Barrezueta, Anna C. Stoll, Renee C. Gentzel, Jonathan A. Sugam, Jacob Marcus, Danny A. Bitton

**Affiliations:** 1 Discovery Informatics, MSD Czech Republic s.r.o., Na Valentince 4, FIVE Building, Prague 5 - Smichov, Prague, 150 00 Czech Republic; 2 Data Science, MSD Czech Republic s.r.o., Prague, Czech Republic; 3 Neuroscience Discovery Merck & Co., Inc., Rahway, NJ USA; 4 Global Software Development, MSD Czech Republic s.r.o., Prague, Czech Republic

**Keywords:** Image registration, Deep learning, Mouse brain

## Abstract

Annotation of multiple regions of interest across the whole mouse brain is an indispensable process for quantitative evaluation of a multitude of study endpoints in neuroscience digital pathology. Prior experience and domain expert knowledge are the key aspects for image annotation quality and consistency. At present, image annotation is often achieved manually by certified pathologists or trained technicians, limiting the total throughput of studies performed at neuroscience digital pathology labs. It may also mean that simpler and quicker methods of examining tissue samples are used by non-pathologists, especially in the early stages of research and preclinical studies. To address these limitations and to meet the growing demand for image analysis in a pharmaceutical setting, we developed AnNoBrainer, an open-source software tool that leverages deep learning, image registration, and standard cortical brain templates to automatically annotate individual brain regions on 2D pathology slides. Application of AnNoBrainer to a published set of pathology slides from transgenic mice models of synucleinopathy revealed comparable accuracy, increased reproducibility, and a significant reduction (~ 50%) in time spent on brain annotation, quality control and labelling compared to trained scientists in pathology. Taken together, AnNoBrainer offers a rapid, accurate, and reproducible automated annotation of mouse brain images that largely meets the experts’ histopathological assessment standards (> 85% of cases) and enables high-throughput image analysis workflows in digital pathology labs.

**Supplementary Information:**

The online version contains supplementary material available at 10.1007/s12021-024-09679-1.

## Introduction

In neuroscience and the study of neurodegenerative disorders, histological methods are widely used to evaluate the pathology present in brain samples of preclinical rodent models of disease (LaFerla & Green, 2012). To provide an impartial assessment of the histopathological aspects of a given preclinical model, evaluation should be as unbiased, non-subjective and reproducible as possible. Consistency and quantitative measurements are therefore essential for modeling the pathology present in brain regions at defined stages of the disease, as well as for monitoring the progression of disease over time (Belfiore et al., 2019; Carrigan et al. 2022). Moreover, quantitative evaluation of histological tissue biomarkers, such as markers of cell death (Fricker et al., 2018), pathological protein accumulation (Spires-Jones et al., 2017), or markers of inflammation (Rauf et al., 2022), are similarly critical for successful assessments.

Historically, skilled pathologists or trained technicians have been employed to manually identify specific regions of interest (ROI) in rodent brain samples and to quantify biomarkers of interest across multiple brain regions (Saravanan et al., 2017). Such manual examination is laborious and subjective, limiting the throughput of the development of preclinical rodent models and the testing of therapeutic targets for neurological disorders. In cases where multiple types of image segmentations and annotations are additionally required, the labor and time taken for the entire analysis may increase even further. Moreover, inherent human errors can be amplified by the rote nature of this manual work.

Opportunities to improve and automate pathological analysis workflows have emerged due to the growing efforts of digitization of histological images and creation of publicly available digital mouse brain atlases. Automated image annotation software tools have the potential to enhance throughput of preclinical histopathological assays, as well as to decrease the subjective evaluation and inherent variability in traditional pathological analysis (Technavio, 2022; Baxi et al. 2022). Such annotation tools may offer automation of the pathologists’ routine, and consequently free more time for data analysis.

At present there are no commercial tools that provide an automated segmentation of histological rodent brain sections. A few academic tools have been developed and published to date, (Xu et al., 2020; Shiffman et al., 2018; Yates et al., 2019), yet they are limited in terms of the types of Whole-Slide histology Images (WSIs) and histological stains they support. As described by Xu et al*.*, existing segmentation models of brain regions are typically based on in-life imaging modalities such as Magnetic Resonance Imaging (MRI) and Computed Tomography (CT) scans, which are at lower resolutions than scanned WSIs. The high-resolution WSIs have discontinuous levels of intensities, preventing the use of segmentation algorithms that rely on the use of homogeneous intensities to identify regions.

In scientific research, it is widely acknowledged that 3D atlases provide superior insights compared to 2D atlases. However, within the pharmaceutical domain, our focus is to assist scientists, who primarily rely on 2D imaging slices in their work. In this context, one of our key objectives is to tackle the challenges associated with artifacts and imperfections that exist in real mouse brain slices, which may deviate from the idealized representations of pristine and symmetrical brains commonly found in encyclopedic resources. There are currently two deep-learning based tools that are publicly available for annotation of 2D images, DeepSlice (Carey et al., 2023) and a more recent study on Spatial Landmark Detection (Ekvall, 2024). *DeepSlice* showcases significant strengths in terms of speed and automation, making it particularly suitable for high-throughput studies. However, it primarily relies on affine registration, which could potentially limit its accuracy in handling complex local distortions.

In contrast the Spatial Landmark Detection tool (Ekvall, 2024)*,* offers an improved precision but a lower throughput, which makes it an ideal choice for smaller-scale studies that require meticulous accuracy.

In addition, segmentation tools that have been developed for non-neuronal tissues or to differentiate tumor from normal tissues have primarily relied on classification of cell subtypes using machine or deep learning Baxi et al. (2022). Such an approach for segmentation of neuronal tissues can successfully detect differences between white and grey matter regions of the brain, but faces greater challenges in separating similar, but spatially differentiated regions, such as differences between cortical regions (Kiwitz et al., 2020; Fainstein et al. 2016) furthermore, most tools that do exist have been trained to focus on a single type of staining, such as Nissl-stained tissue sections or hematoxylin and eosin (H&E) stains, while only a few can accommodate chromogenic and fluorescent immunohistochemical (IHC) assays.

Here we present AnNoBrainer, a novel pipeline for an automated annotation of mouse brain digital pathology 2D images that supports multiple types of Whole-Slide histological Images and histological stains. AnNoBrainer is designed to address cases in which a single large Whole-Slide includes multiple individual brains that require separate annotation. This allows for automated analysis at scale, particularly when there is a need to analyze a high throughput of slides. Each individual brain present in the slide is registered against template ROIs of interest. AnNoBrainer combines murine brain detection using mask region-based Convolutional Neural Network (mask R-CNN) and an automated brain labelling with user-defined experimental metadata. Moreover, the pipeline incorporates a deep learning classifier for optimal matching of detected brains with brain atlas layers. Furthermore, AnNoBrainer enables accurate non-linear registration enhanced by landmarks-based regularization. Compared to existing deep-learning based approaches (Carey et al., 2023; Ekvall, 2024) AnNoBrainer offers an easy deployment, fully automated and high-throughput analysis with comparable accuracy, making it suitable for an industrial-scale annotation of large volumes of digital pathology slides.

AnNoBrainer offloads Pathologists’ repetitive work to a machine and approximately halves the time spent on image annotation while maintaining manual annotation standards. Finally, AnNoBrainer not only streamlines image analysis workflows, but also increases the throughput of digital pathology studies in a pharmaceutical setting. AnNoBrainer is freely available at https://github.com/Merck/AnNoBrainer.

## Methodology

### Data Collection

Data from a study that characterized the aSyn pre-formed fibrils surgical model of synucleinopathy in A30P transgenic mice were used for model validation (Schultz et al. 2018). Image selection was largely dependent on stain quality and tissue integrity. Significant tears and deformation of the tissue that can dramatically affect detection of morphological boundaries were excluded. Likewise, images were excluded where stain quality was not sufficient for detection of morphological boundaries, or it was substantially different from the staining intensity of neighboring sections. The initial dataset consisted of 313 brains samples on 19 slides. AnNoBrainer was tested eventually on only 229 brain samples, since some brain samples did not pass the initial quality control e.g., due to processing artifacts (e.g., missing half of a brain). Selected brain samples on each slide were annotated by expert neuroscientists and were limited to three different brain regions as follows: CP (caudioputamen), SNr (substantia nigra) and PAG+AQ (periaqueductal gray + cerebral aqueduct). The final dataset for quantitative analysis consisted of 89 brains samples from 17 different layers for CP, 105 brains samples from 18 different layers for SNr and 35 brains from 7 different layers for PAG+AQ. The distribution of brain section in each layer within each region is shown on Supplementary Fig. S2.

The primary objective of our study was to conduct a qualitative and quantitative analysis of AnNoBrainer, which necessitates both the registration results and corresponding ground truth annotations. Manual annotation of brain regions can be challenging for individuals without specialized training in neuroscience. Thus, we sought the assistance of expert neuroscientists to provide ground truth annotations. Our ground truth dataset only encompasses annotations for the three regions that were specifically investigated in our experiments. While this might seem at first as a limitation, we demonstrate the ability of AnNoBrainer to annotate every region of interest in the brain sample (Fig. S1).

We also conducted a qualitative analysis of 7 additional brain regions of interest (ROIs) commonly used in neuroscience, including ACB (Nucleus Accumbens), aco (Anterior Commisure), cc (Corpus Calosum), HY (Hypothalamus), CTX (Cerebral cortex), LSr (Lateral septal nucleus – rostral part), MS (Medial septal nucleus) and HIP (Hippocampus region). These regions cover a significant portion of the brain.

### Manual Annotations

Manual annotations of the caudoputamen (CP) had been made based on morphological boundaries. The lateral edge of the CP was delineated by the corpus callosum and the medial edge by the lateral ventricle. The dorsal CP was separated from the ventral CP by drawing a horizontal line from the most ventral aspect of the lateral ventricle, where it often meets the anterior commissure, across to the lateral edge of the CP. The full striatal area was not captured manually for two reasons. First, the site of injection into the CP only targeted the dorsal portion of the CP. Secondly, accurately, and quickly discerning the ventral CP from the nucleus accumbens using a hematoxylin stain is more challenging than identifying the lateral and medial boundaries of the CP. Using only well-defined morphological markers for manual annotations enabled consistency and minimized biases in drawing the region of interest. However, this also illustrates why manual annotations often sacrifice capturing the full anatomical region of interest in favor of accommodating technical ease.

### Qualitative Analysis

In addition to manual annotations, which can be time-consuming and impractical for every brain region, we conducted a qualitative analysis of additional brain regions (see Data Collection section). During the evaluation, each annotation was rated independently by two expert neuroscientists on a scale from 1 to 5 representing the need for manual adjustment of the automated annotation. In category 1 annotations required no manual adjustments, in category 2 required only minor adjustments, in category 3 medium adjustments were needed, for category 4 annotations required significant manual adjustments, and in category 5 the ROI was completely mis-annotated. This qualitative analysis proved to be too granular as it reflected disagreement between the expert annotators, highlighting the challenges in annotating brain images even by experts. We therefore grouped these data into two categories “useful” and “not useful”. Annotations falling into category 1 (no manual adjustment) or category 2 (minor adjustment) were considered "useful", as these instances required minimal effort and indicated a fairly accurate annotation. Annotation rated 3 and above were considered "not useful" as they required significant adjustments, raising concerns about their accuracy and reliability.

### Allen Mouse Brain Atlas

The Allen mouse brain atlas (Lein et al., 2007) consists of 132 coronal sections evenly spaced at 100 µm intervals and annotated to detail numerous brain regions. It was designed to be easily integrated into digital applications in the field of automated histological segmentation, and it is considered reliable source of mouse brain anatomy delineating regions of interest, which are mapped and labeled.

### Brain Detection

Detection of brains and hand-written notes on a slide is considered an object detection problem, which consists of two subproblems: (i) detecting the objects of interest (brains and handwritten notes) and (ii) segmenting the image with bounding box detection to label the category. For this purpose, a transfer learning strategy (Zhuang et al., 2021; Talo, 2019) was followed, and a pre-trained mask R-CNN model (He et al., 2017) was modified by replacing its last fully connected layer with a set of new fully-connected layers retrained on 20 manually labeled slides aiming to detect two distinct categories – 1) mouse brains and 2) handwritten notes. For the training process, ADAM (Adaptive Moment Estimation) optimizer was used with 60 epochs and batch size of 2. Following brain detection, bounding boxes were generated for each individual mouse brain on a slide and centroids were calculated to form a brain centroid grid.

### Linking of Detected Brain Images

To differentiate between experimental and control group brains, an experimental table was manually created for each slide. To link the detected brains from the slides to their corresponding descriptions in the experimental table, the following approach was employed. First, a brain centroid grid from the brain detection step was obtained. A similar grid was then constructed from the experimental table using its row and column coordinate structure. Both grids were then standardized to ensure that they were in the same unit of measurement. To connect the corresponding points on each grid, Hungarian algorithm (Kuhn, 2005) was applied. The algorithm assigns each pair from both tables in such a way that the total sum of distances is minimized.

### Matching with Reference Atlas Layer

Matching of brains to their respective reference (z-slice) layers is a manual, non-trivial and subjective task that requires expert knowledge. Multiple convolution neural networks architectures for classification were trained and tested, using a standard test/train split approach and image augmentations, including rotation, random brightness, channel flip, median blur, and elastic transforms (Buslaev et al., 2020). The latter is an important component of the training since individual brains are cut by hand and a certain level of asymmetry is generally present. The ResNet34 (He et al., 2015), and Efficient Net architectures were used (Tan & Le, 2020). Since this task can be also interpreted as a regression problem, a Label Smoothing Cross-Entropy Loss function (Müller et al., 2020) were used for the training. The training used pre-trained models on the ImageNet dataset and only replaced the last fully connected layers that are responsible for the classification itself. These were fully trained from scratch using a batch size of 12 and ADAM optimizer for 12 epochs.

### Image Registration Process

Image registration is an iterative optimization process that searches for best suitable geometrical transformation to spatially align two images, a reference image, and a target image. The image registration process implemented in our pipeline consists of subsequent steps of geometric alignment between reference Allen mouse brain atlas layer and mouse brain identified on a slide.

Airlab Library (Sandkühler et al., 2020) and the detection of sparse cross-domain correspondences (Aberman et al., 2018) were the main tools utilized for AnNoBrainer’s custom registration pipeline. AirLab provides flexibility with various image registration methods, loss functions, and regularization techniques all using Pytorch, and can be run on a GPU cluster. Affine Registration was the first step in our procedure followed by Elastic Registration to correct local nonlinear disparities. The objective function for image registration was extended by considering a sparse correspondence detected in target image and in the template image via (Aberman et al., 2018). Thereafter, the elastic registration regularization term was extended to minimize the distance between the corresponding landmarks while ensuring smooth geometric transformation.

### Affine Registration

Affine registration is a technique used to align two images by applying 2D linear transformations and translations. These transformations involve operations like scaling, reflection, and rotation. The objective is to gradually transform the registered image to closely resemble the template image, minimizing the loss function. To preserve the original shape of the image, similarity transformation is utilized, involving simple operations like rotation and reflection. The affine registration process employs the Normalized Cross-Correlation loss objective function. Airlab's backend, which enables iterative GPU-based optimization, is used for the registration. The optimization software gradually adjusts the registered image to maximize its similarity to the template image. The ADAM optimizer is utilized with a learning rate of 0.01 and 1000 iterations.

### Elastic Registration

Following the affine registration alignment, the elastic registration process takes place. Elastic registration estimates a smooth, continuous function to map points from the registered image to the template image. There are three commonly used transformation models: linear/dense, non-linear/interpolating, and dense models. AnNoBrainer employs the non-linear/interpolating approach. In this approach, to transform a point x in the image, the displacement f(x) is interpolated from neighboring control points using a basis function. A B-spline kernel is used as the basis function. The diffeomorphic approach is employed to maintain topology preservation. Additionally, a regularization term is introduced to the affine registration to limit the number of sampling points. The displacement field is subjected to a Regularizer (DiffusionRegularizer) that penalizes changes in the transformation. Similar to the affine registration, the normalized cross-correlation loss function is used for optimization. The Adam optimizer is employed with a learning rate of 0.01 and 1000 iterations for optimizing the transformation field.

### Landmark Regularization Term

During the registration process, two common edge cases arise: excessive or insufficient distortion. These cases are typically a result of balancing the loss function and regularization term. To achieve realistic and anatomically reasonable distortions, a smaller distortions approach with higher regularization is commonly used. However, a common issue arises when the tissue cut is not perpendicular to the z-axis of the brain, leading to anatomical differences between the brain hemispheres and imperfect alignment in some areas, especially when higher regularization is used. To address this, a higher level of flexibility is necessary. Consequently, an additional step was implemented to adjust the regularization term for specific parts of the image, allowing for higher distortion locally. Image landmarks play a crucial role in this process. In image processing, landmarks are characteristic features or structures in an image. In the context of this work, landmarks are points at the same semantic locations across two images, such as the left edge of an organ. When the landmarks are aligned, the regularization term behaves as it would without landmarks. However, when there are significant differences between the landmarks of the template image and the registered image, it locally reduces the effect of the regularization term. This approach leads to potentially improved overall image alignment. To identify these landmark points, a pre-trained neural network from Neural Best-Buddies: Sparse Cross-Domain Correspondence (Aberman et al., 2018) was utilized. This neural network helps find semantically matching areas across two cross-domain image pairs (across two brain samples in our case). Then, a regularization term derived from the landmarks was constructed. The objective was to minimize the squared Euclidean distance between corresponding landmarks. The squared Euclidean distance was preferred over the classic square root version as it preserves the smoothness of the function at all points, which is crucial for gradient optimization.

A Landmark regularization term ($LRT$):

$${d}^{2}\left(p, q\right) = \sum\limits_{ n=1}^{ N}\sum\limits_{ d=1}^{ D}{\left({p}_{n,i}-{q}_{n,i}\right)}^{2}$$

where $p$ denotes warped landmark point from moving image, $q$ denotes landmark point from fixed image, $N$ represents number of landmark point and $D$ represents a dimension corresponding to x and y position in image.

An elastic Regularization loss uses Normalized Cross Correlation ($NCC$) extended with a Diffusion Regulariser term ($DF$). Thus, the loss function extended with a Landmark regularization term is defined as follows:

$$Loss=NCC+{\lambda }_{1}DF + {\lambda }_{2}LRT$$

where terms ${\lambda }_{1}$ and ${\lambda }_{2}$ are both selected as 0.5.

## Results

### AnNoBrainer Features Seamless Mouse Brain Annotation Using Deep Learning

**Fig. 1:**
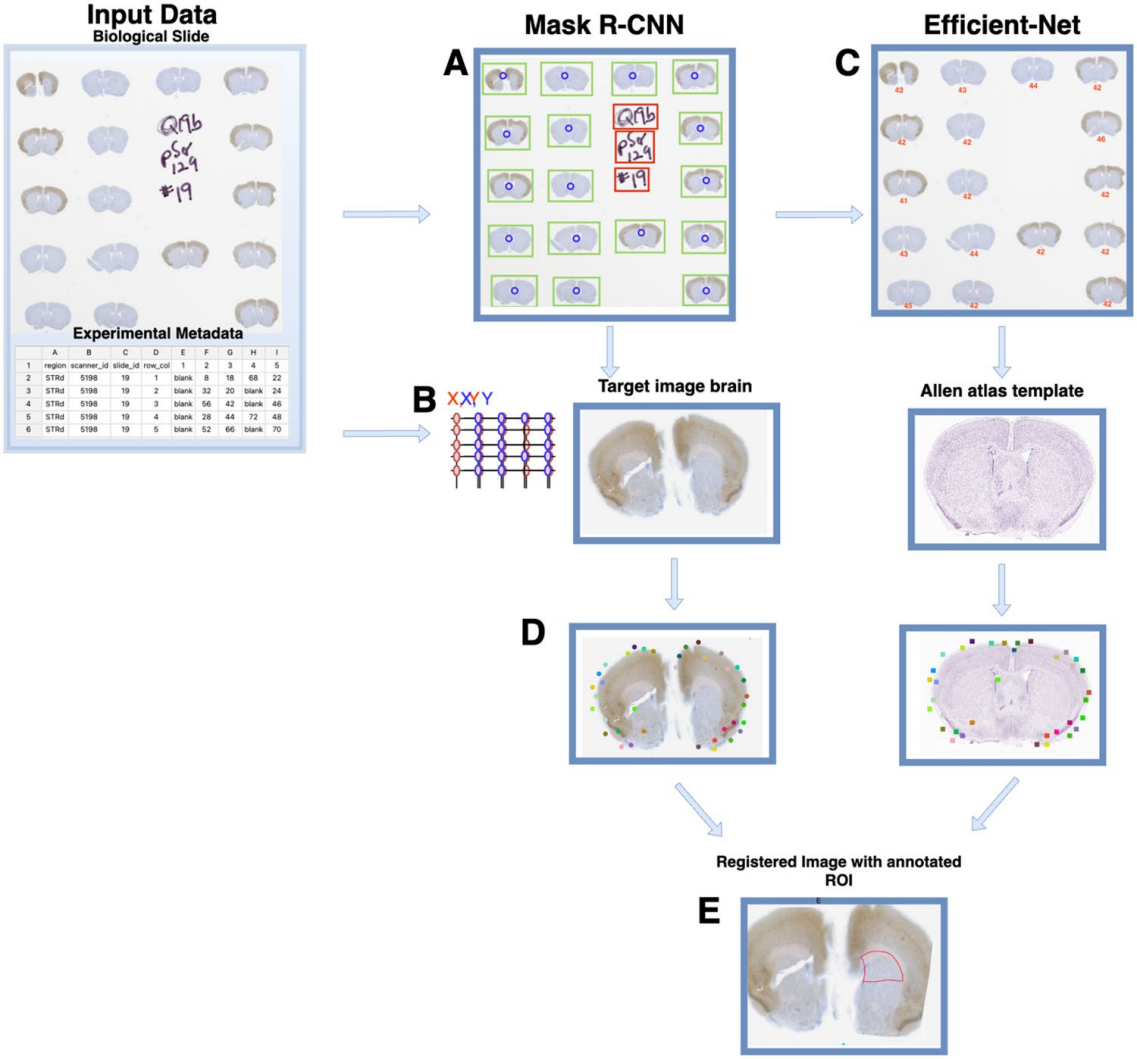
AnNoBrainer enables an automated identification of brain tissue from multi-brain slides, their matching to experimental metadata and reference brain templates as well as efficient and robust image registration. **A** a mask R-CNN identifies brains and background handwritten notes **B** an Hungarian algorithm is used to link experimental metadata with individual brains **C** Efficient-Net model is used for matching a target brain with its corresponding Allen atlas layer **D** image registration of brain with Allen atlas template using affine and elastic registration that is enhanced by custom landmark regularization **E** region of interest is geometrically transformed to target brain

Histopathological assessment of preclinical murine models is instrumental for neurodegenerative studies, yet it is largely dependent on manual image analysis and annotation by experienced pathologists, which is time-consuming, subjective, not consistent, or scalable. To fill in this gap AnNoBrainer offers an end-to-end pipeline that significantly accelerates digital pathology campaigns. The pipeline is comprised of four main steps (Fig. 1), as follows. First, the tool detects individual brain sections present on the 2D slides using mask R-CNN deep learning model that also efficiently identifies and disregards noise on the physical slides such as hand-written notes (Fig. 1A). Second, using Hungarian algorithm (Kuhn, 2005) AnNoBrainer automatically labels individual brains with user-defined experimental metadata provided in an Excel spreadsheet format (Fig. 1B). Thereafter, a deep learning classifier is employed to identify the optimal Allen mouse brain atlas template for each brain (Fig. 1C). Once the reference layers for all individual brains are identified, image registration for each image pair is taking place to determine optimal transform parameters (Fig. 1D). Subsequently, user-selected ROIs are transferred from the reference atlas layer to the target brains ROIs (Fig. 1E).

### AnNoBrainer Exhibits Flawless Detection of Brain and Background Objects

Often, in a digital pathology laboratory, murine brain sections from multiple individuals are placed on a single slide and typically accompanied by hand-written notes. Therefore, the annotation pipeline must identify and distinguish between brains and background objects. For this task, AnNoBrainer employs a pre-trained mask R-CNN model (Table 1) that was further extended to include two designated ‘brain’ and ‘note’ categories. The model was then fine-tuned to distinguish between these two new classes. Briefly, for training purposes 20 slides were manually annotated with bounding boxes representing the respective two new classes. To ensure that the model will be able to generalize effectively in the future, the slides for the training were selected in such a way that they were highly variable and represented a broad spectrum of use cases, as follows. Labelled slides accounted for instances with randomly missing brains, rotated and torn tissue, and instances with many or no hand-written notes at all.

**Table 1:** Deep learning models used in this study

Model Type	Function	Input	Output
Mask R-CNN	Detects individual brains and handwritten notes present on the slide	Digital Slide image	Coordinates of individual brains present on the slide
EfficientNet-B0	Matches brain to the most similar Allen brain atlas reference layer	Individual brain image	Predicted Z-axis from the Allen brain atlas

The fine-tuned mask R-CNN model was then applied to 20 new slides, where it perfectly identified and segmented all individual brains on each of the slides as well as correctly classified all hand-written note objects.

### AnNoBrainer Efficiently Links Detected Brain Images to Experimental Metadata

Experimental metadata associated with the brain images are critical for downstream data analysis. AnNoBrainer enables seamless matching between user provided metadata (Excel table format) and their respective brain images. Briefly, the Excel table containing the metadata is first converted to a coordinate grid (Fig. 1B, red). Once individual brains are detected on a slide, their centroids are computed to derive a second grid (Fig. 1B, blue). Both grids are then aligned via standardization. Euclidean distance between all points in both grids is computed and Hungarian algorithm is employed to assign each brain with its associated unit grid node.

### AnNoBrainer Matches Brain Images to Reference Brain Atlas Templates With High Accuracy

Correct classification of brain layers is crucial for experimental neuroscience given the spatial gene expression patterns across different layers and cell types. Databases such as the Allen mouse Brain Atlas provide a comprehensive anatomical brain map that can be integrated into histopathological analysis workflows. Although matching brain layers to detected brain objects can be performed manually by an expert pathologist, it is not a trivial task and is somewhat subjective. AnNoBrainer offers expert users to either manually select the corresponding reference brain template for each brain image or alternatively to use an automated matching approach. For the latter, it employs a deep learning model trained on labelled data that were manually curated by expert pathologists. To ensure variability within the training set, images were selected in all shapes and forms, including clean and symmetrical brains alongside asymmetrical and slightly torn brain sections. Similarly, images used for training were resized to 460 pixels to ensure consistency. Importantly, training data only accounted for a subset of atlas z-slices (39–73 CP, Fig. S3), each containing expert-labelled images, limiting the prediction of templates to this spectrum of z-slices. Selected examples of labelled images for reference brain atlas training are shown in Fig. S4. Multiple Convolutional Neural Network (CNN) were initially tested using standard data train-test split and image augmentation (e.g., brightness, blur, rotation etc.,) fine-tuned to the extent that training and testing losses ceased to decrease any further. The EfficientNet-B0 model outperformed the ResNet-34 model at default or fine-tuned states, reaching an overall 94% accuracy level when a misclassification tolerance of 1–2 neighboring layers with respect to expert annotation was applied (Tables 1 and 2). This result ensures relevant matching of brain images against their respective template, providing sufficient accuracy for an automated workflow.

**Table 2:** Model performances for brain atlas layers matching

Method	Exact accuracy	± 1 accuracy	± 2 accuracy
Random	7%	18%	28%
Resnet 34 (default)	40%	73%	88%
Resnet 34 (tunned)	50%	80%	91%
EfficientNet-B0	59%	86%	94%

### AnNoBrainer Offers a Robust Image Registration Technique That Ensures Optimal Spatial Alignment

Once a 2D reference brain template is identified it needs to be spatially aligned to its respective physical brain image on the slide via an iterative optimization process known as image registration. AnNoBrainer employs robust, non-linear image registration techniques that combines Affine and Elastic registration approaches for geometric image alignment and smoothing, respectively. To further improve image alignment, AnNoBrainer incorporates an optional automated detection of semantic locations on the images that essentially act as anchor characteristic features between the image pair, also referred to as custom landmark terms (Fig. 1D).

The performance of the automated registration was evaluated using a quantitative and qualitative analyses. For the quantitative analysis, a segmentation metric between the expert annotated ROIs and the ROIs automatically registered by AnNoBrainer. In a semantic segmentation the aim is to predict a mask. Put differently, to predict where the actual object of interest is present. Here, both the expert annotated, and the automatically registered ROIs were treated as a binary mask i.e. 0 and 1. Thereafter, F1 score was used to quantitatively assess the registration accuracy by measuring the similarity between expert-annotated and predicted ROIs for each image pair. Furthermore, the effect of custom landmarks terms on registration quality was also evaluated.

**Fig. 2:**
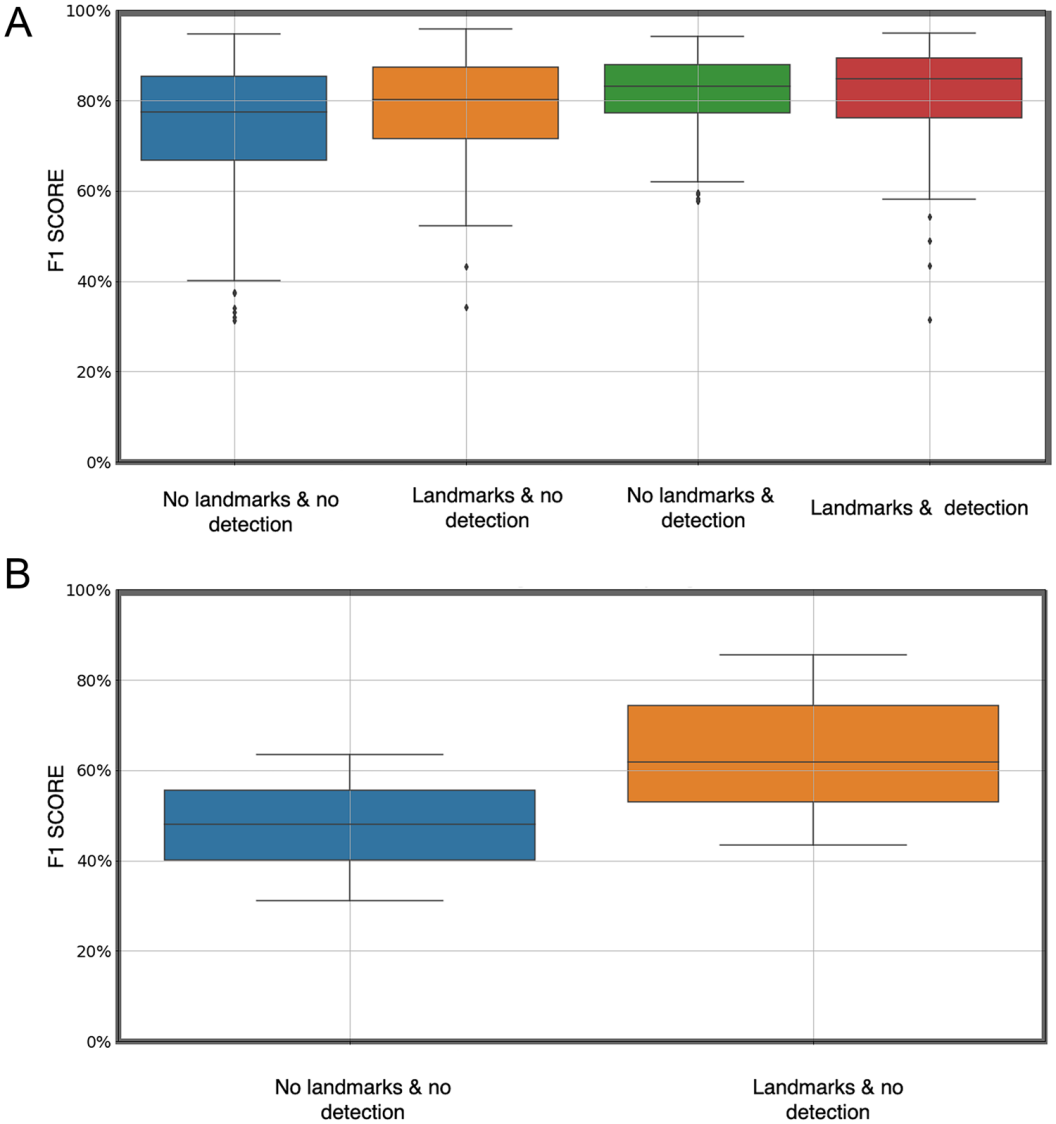
Registration accuracy for CP and PAG regions respectively. **A** registration accuracy for CP region with various pipeline settings, as indicated on the x-axis. **B** registration accuracy for PAG region with various pipeline settings, as indicated on the x-axis

Regardless of the brain region tested (Caudoputamen - CP, Periaqueductal gray - PAG, and Substantia nigra - SNr) image registration quality was significantly improved when AnNoBrainer automated atlas layer detection was combined with custom landmark terms, compared to manual layer selection by expert without the use of landmark terms (Fig. 2A, B, t-test p < 0.00013).

**Fig. 3:**
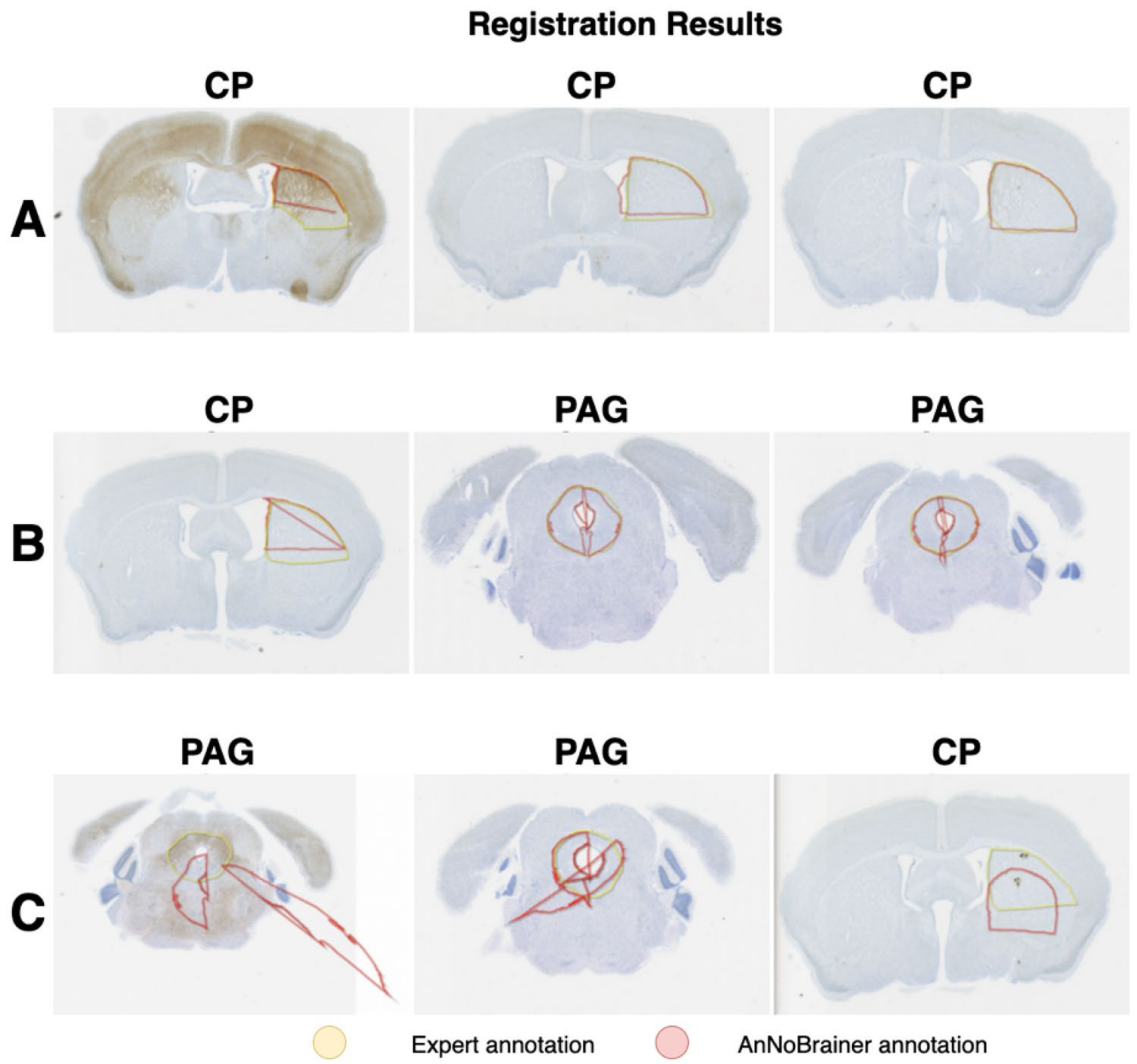
Registered ROI’s by AnNoBrainer. **A** Satisfactory annotation not requiring manual adjustments, **B** Satisfactory annotation requiring slight manual adjustments, **C** Unsatisfactory annotation requiring significant manual adjustments. These images represent only a subset of the data, providing examples of the final annotation by AnNoBrainer compared to annotation by expert pathologists

Finally, a comparison between manual annotation and expert annotation was performed revealing that the majority (> 67%) of AnNoBrainer automated annotations met the expert annotation standards while some (~ 18%) needed minor adjustments (Fig. 3A, B). In approximately 15% of the cases AnNoBrainer completely failed to precisely annotate the brain images within accepted boundaries, primarily due to discrepancies in structural information between the input data and the Allen brain atlas (Fig. 3C).

### AnNoBrainer Can Support Annotation Across Numerous Brain Regions

**Fig. 4:**
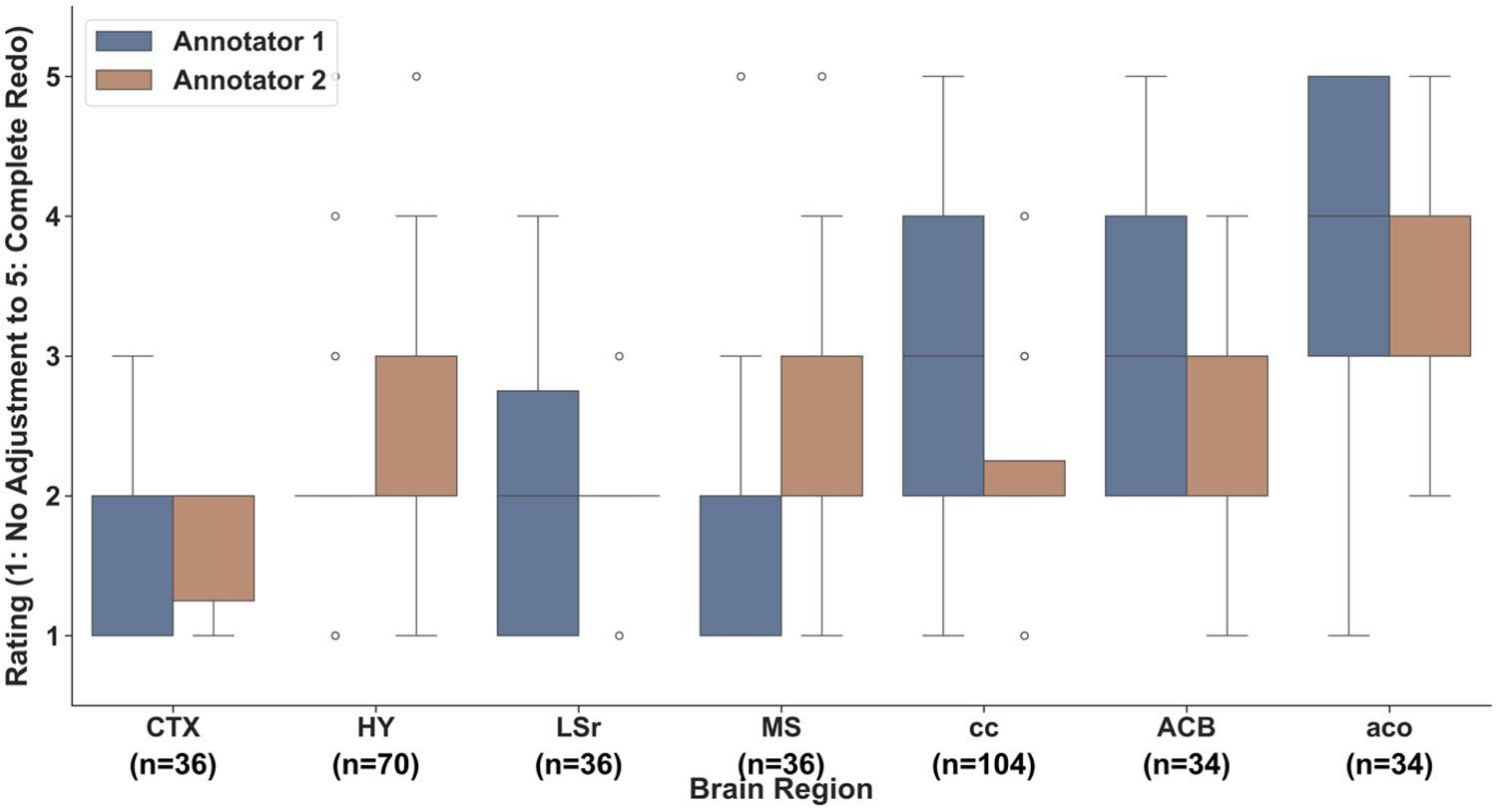
Qualitative assessment of AnNoBrainer annotations of pathologically relevant ROIs. Two expert neuroscientists independently evaluated the annotation quality of AnNoBrainer on 7 selected ROIs across 52 brains as indicated (‘n’ denotes the number of registered regions). The agreement between the two experts was evaluated using Kappa statistics. A Kappa score of 0.17 indicates a slight correspondence between the two annotators (within the 0.01 to 0.20 range for slight agreement)

To assess the ability of AnNoBrainer to annotate other commonly used regions in neuroscience, we conducted a qualitative analysis of additional 7 ROIs across 52 brain samples. Where multiple ROIs were registered by AnNoBrainer, and annotation quality was evaluated independently by two expert neuroscientists. The expert annotators were asked to rate the quality of annotation on a scale of 1–5 based on the adjustment level required to accurately annotate the respective ROI. Where 1 indicates accurate annotation with no adjustment needed and 5 denotes complete mis-annotation (Fig. 4). Evaluation of the correspondence between the two expert annotators using Kappa statistics revealed a Kappa score of 0.17, indicating only a slight agreement between the experts. These results highlight the inherent challenges and potential variability when different experts interpret annotation of brain ROIs, underlining the necessity for more standardized and reproducible methods in ROI annotation. These findings emphasize the need for an automated annotation tools to reduce the subjectivity and variability associated with manual annotation.

**Fig. 5:**
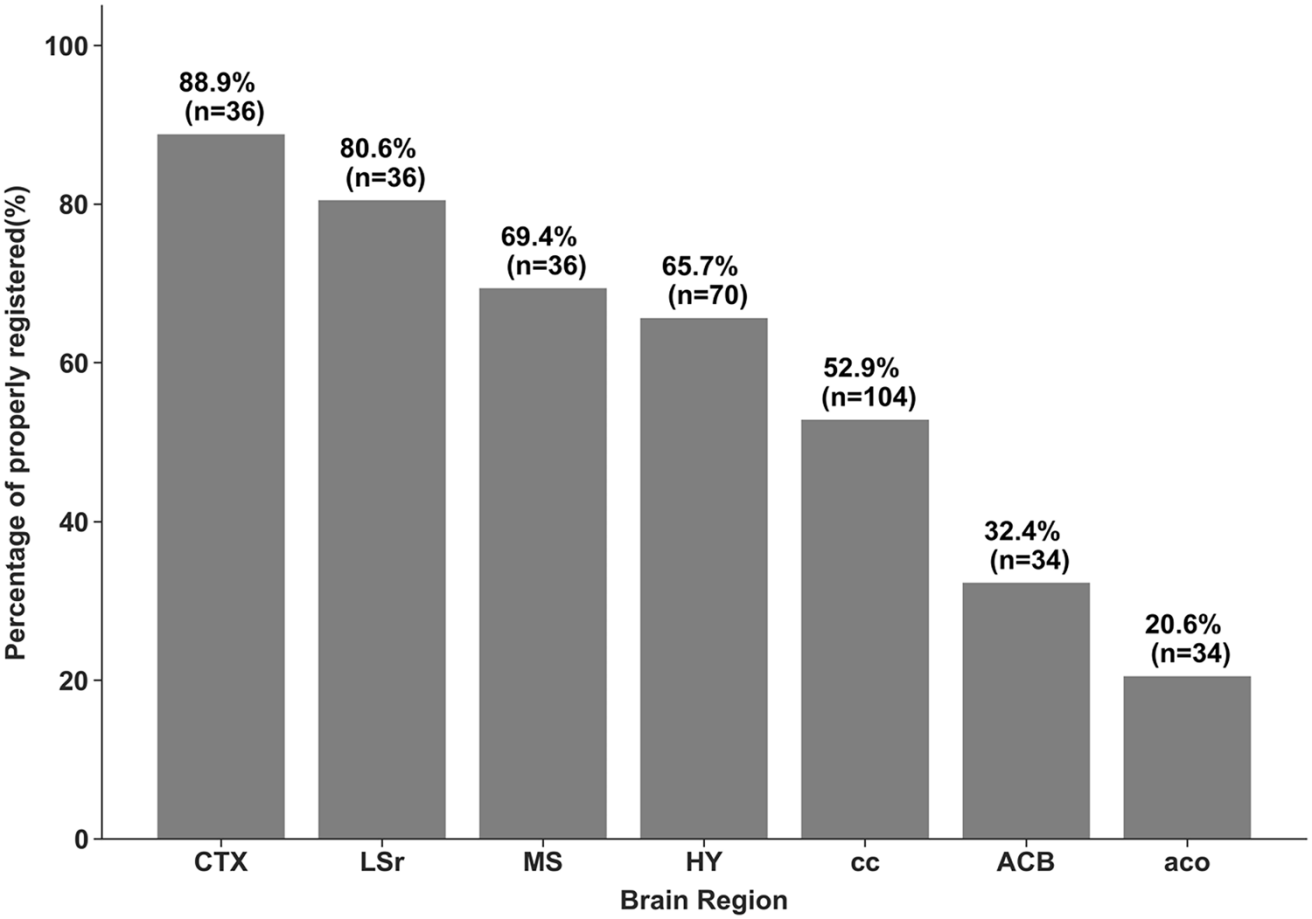
AnNoBrainer’s performance of annotated regions of interest (ROIs) based on evaluation performed by two experts. Annotation was classified “useful” or “not useful” based on the adjustment level needed to accurately annotated the entire ROI, where only minor or no adjustment levels were considered useful. The percentage of successfully annotated ROIs for various regions are as shown. The regions CTX, LSr, MS and HY exhibit a particularly high percentage of successful registrations. Kappa statistics score was 0.38, indicated fair agreement between the two expert annotators

To highlight the ability of AnNoBrainer to assist pathologists with annotation of various ROIs, we considered only categories 1–2 as useful, where minor or no adjustments were needed (Fig. 5). The remaining annotations were considered “not useful”. A Kappa score of 0.38 reflected a fair agreement between the two experts based on these two categories. Association between the size of the anatomical region of interest and the quality of annotation was observed. Larger regions including CTX, LSr, MS, HY exhibited high percentage of useful annotations (> 65%), while smaller regions including cc, ACB and aco displayed a lower number of useful annotations. These results may reflect the difficulties in annotating small regions that typically exhibit intricate details and subtle differences, making their alignment more prone to errors by expert annotators, let alone by automated annotation tools. In addition, it may also raise that possibility that different staining techniques could affect the annotation accuracy due to a reduced visibility of distinctive borders between neighboring anatomical regions.

We also demonstrate the ability of AnNoBrainer to annotate brain samples of varied qualities by providing full images of annotated brains (Fig. S1). As expected, AnNoBrainer performed better on brain samples of a higher quality (e.g. without missing tissues, tears, or processing artifacts). Similarly, we provide a complete image output of AnNoBrainer to demonstrate its ability to annotate multiple ROIs across the brain simultaneously (Figs. S5 and S6).

Taken together, AnNoBrainer can significantly aid in the annotation process, improving efficiency, consistency, and throughput of brain image annotation across numerous ROIs.

## Discussion

Pre-clinical neuroscience research relies largely on histopathological analysis of brain samples of rodent models of disease. The emergence of digitized pathology slides and advanced machine and deep learning methods opens new opportunities to automate, streamline and scale up laborious analysis workflows without compromising the quality achieved by human experts.

AnNoBrainer has been successfully applied in multiple high-throughput pre-clinical neuroscience studies in the industry, and it performed well on a large number of slides with a varying quality of brains (e.g., slightly torn, asymmetrical etc.), different stains (e.g. H&E, Nissil, IHC), background noise and various templates. Apart from the benefit of automation of mundane work, AnNoBrainer also reduces the annotation time of a given slide by approximately 50%.

Despite these advantages it is also important to note AnNoBrainer’s limitations. The atlas layer identification model in its current form is only applicable to a limited number of brain slides (z-direction), since it was trained only on slides within the CP region. This limitation can be readily addressed by retraining the model with an extended labelled dataset. Another caveat is that the image registration quality of AnNoBrainer may be affected by the quality of the input brains with respect to torn or missing tissue, where asymmetry or tears found in close proximity to the registered region may result in poor registration around these areas. Additional drawbacks that are associated with the matching of brain layer template and the registration process can be attributed to the discrepancies between the varying layer sizes in the Allen brain atlas and the actual size of the brain slices provided as input. Similarly, certain IHC staining techniques such as DAPI are unlikely to result in high quality registration due to data sparsity or poor morphological correspondence with the H&E staining used in the Allen brain atlas. Important to note that to date Nissl, H&E and some IHC stains (e.g. α-synuclein) were successfully tested, yet AnNoBrainer was designed to also support multiplexed stains and can incorporate other IHC stains too, therefore can support a broader range of studies compared to previously published methods (Kiwitz et al., 2020; Yates et al., 2019) that focus primarily on traditional histology stains. It is likely that different staining techniques may influence the visibility of specific areas of interest and consequently affect the quality of annotation.

Our qualitative analysis clearly indicated that AnNoBrainer improves brain annotation in terms of efficiency, reproducibility, and throughput across different ROIs. However, it also exposed an additional limitation of the tool with respect to annotation of smaller regions that are notoriously difficult to annotate even by expert pathologist.

## Conclusion

In this study we developed an automated, deep learning-based pipeline for mouse brain annotation. Our pipeline can efficiently identify and distinguish mouse brain from noise, link it to its respective experimental metadata and register it with its corresponding brain layer template. AnNobrainer was designed as a modular and extensible annotation pipeline to allow retraining of models and/or introduce further improvements by the community as more labelled data become available or when other staining techniques are preferred.

## Information Sharing Statement

Data sharing is not applicable to this article as no new data were created in this study. Reference brain templates from Allen Brain Atlas (https://mouse.brain-map.org/static/atlas) were used for template matching and data from (Schultz, et al. 2018) were used for model validation. All code for this publication is available in the following GitHub repository: https://github.com/Merck/AnNoBrainer.

## Supplementary Information

Below is the link to the electronic supplementary material.

Supplementary file1 (DOCX 6572 KB)

## Data Availability

All code for this publication is available in the following GitHub repository: https://github.com/Merck/AnNoBrainer.
