# Supplementary material for: AnNoBrainer, An Automated Annotation of Mouse Brain Images using Deep Learning
Source: Neuroinformatics. 2024 Aug 7;22(4):719–30. doi: 10.1007/s12021-024-09679-1 (PMC11579091; doi:10.1007/s12021-024-09679-1)
Supplement: Supplementary file 1 — Supplementary file1 (DOCX 6572 KB) [file 12021_2024_9679_MOESM1_ESM.docx]

**AnNoBrainer, an Automated Annotation of Mouse Brain Images using Deep Learning**

Roman Peter, Petr Hrobar, Josef Navratil, Martin Vagenknecht, Jindrich Soukup, Keiko Tsuji, Nestor X. Barrezueta, Anna C. Stoll, Renee C. Gentzel, Jonathan A. Sugam, Jacob Marcus, and Danny A. Bitton

**Supplementary Material**

**
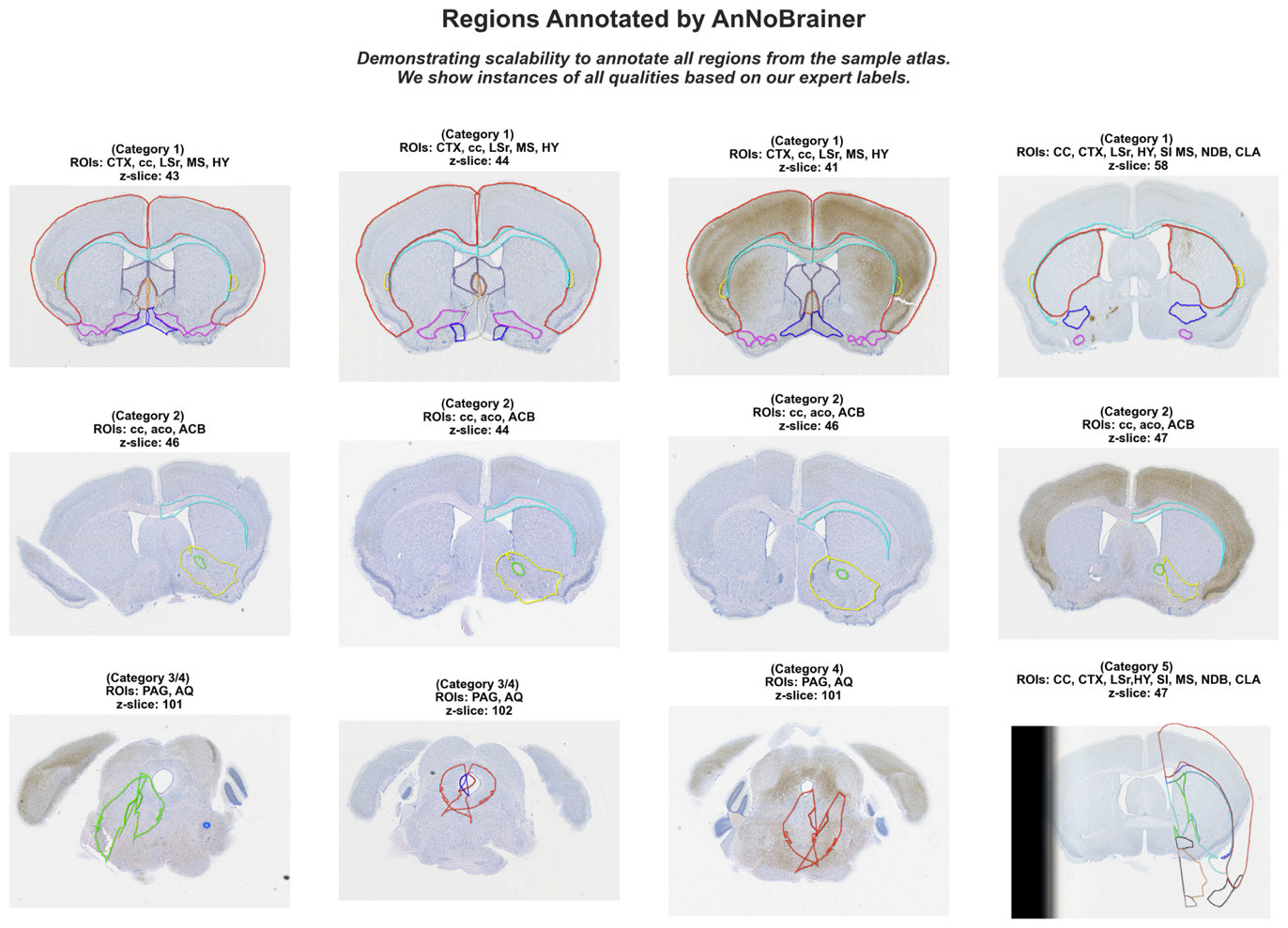
**

**Figure S1**. Regions annotated by AnNoBrainer to demonstrate its scalability to annotate other regions for which expert annotations were not available. The score shown at the top of each brain sample represents the worst ROI score.


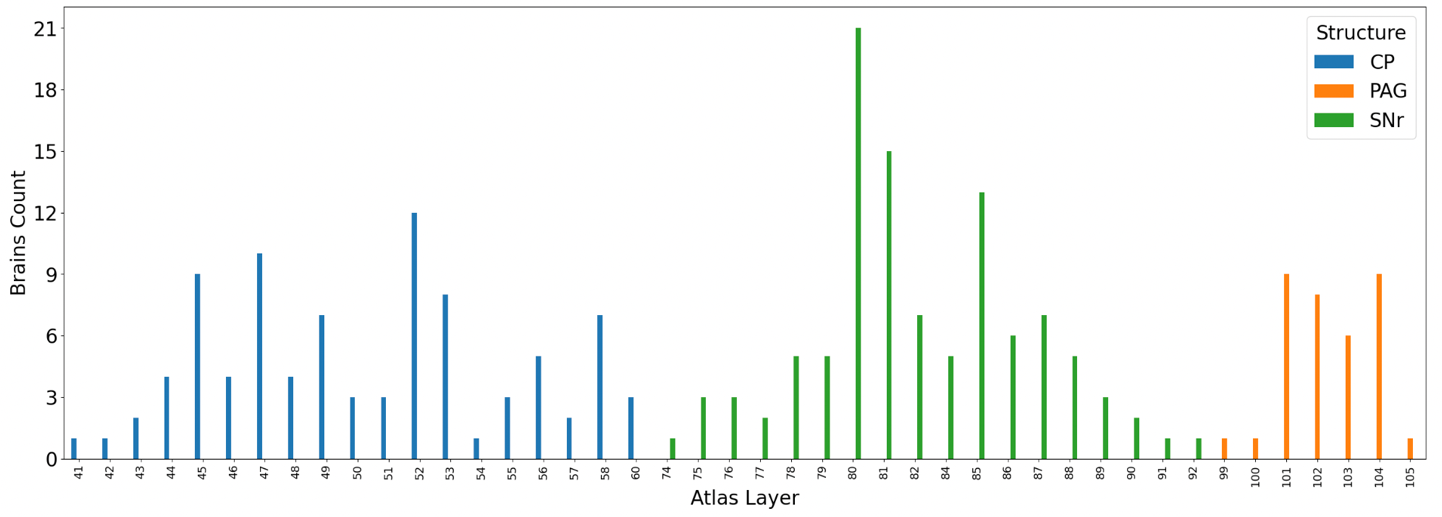


**Figure S2.** Dataset for validation of our pipeline. It shows number of brains in the dataset depending on Atlas Layer number, which is taken from the Allen Mouse Brain Atlas. Different regions in our dataset are distinguished by the color of the bar.

#
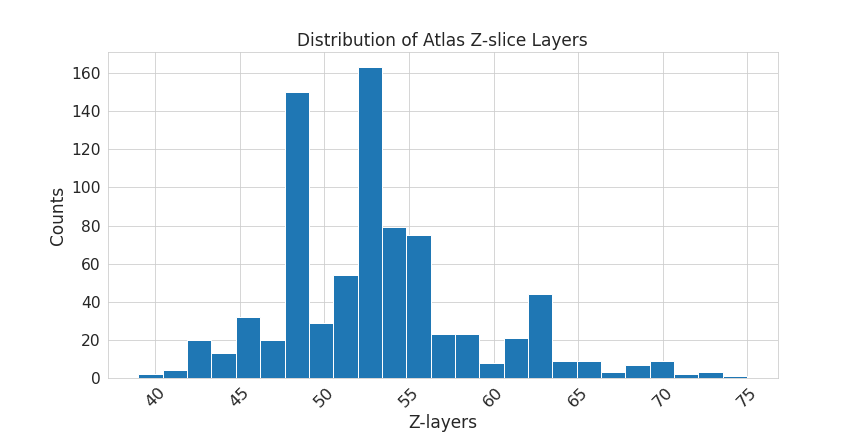


**Figure S3**. Distribution of atlas slices used for training of the brain to layer matching models.


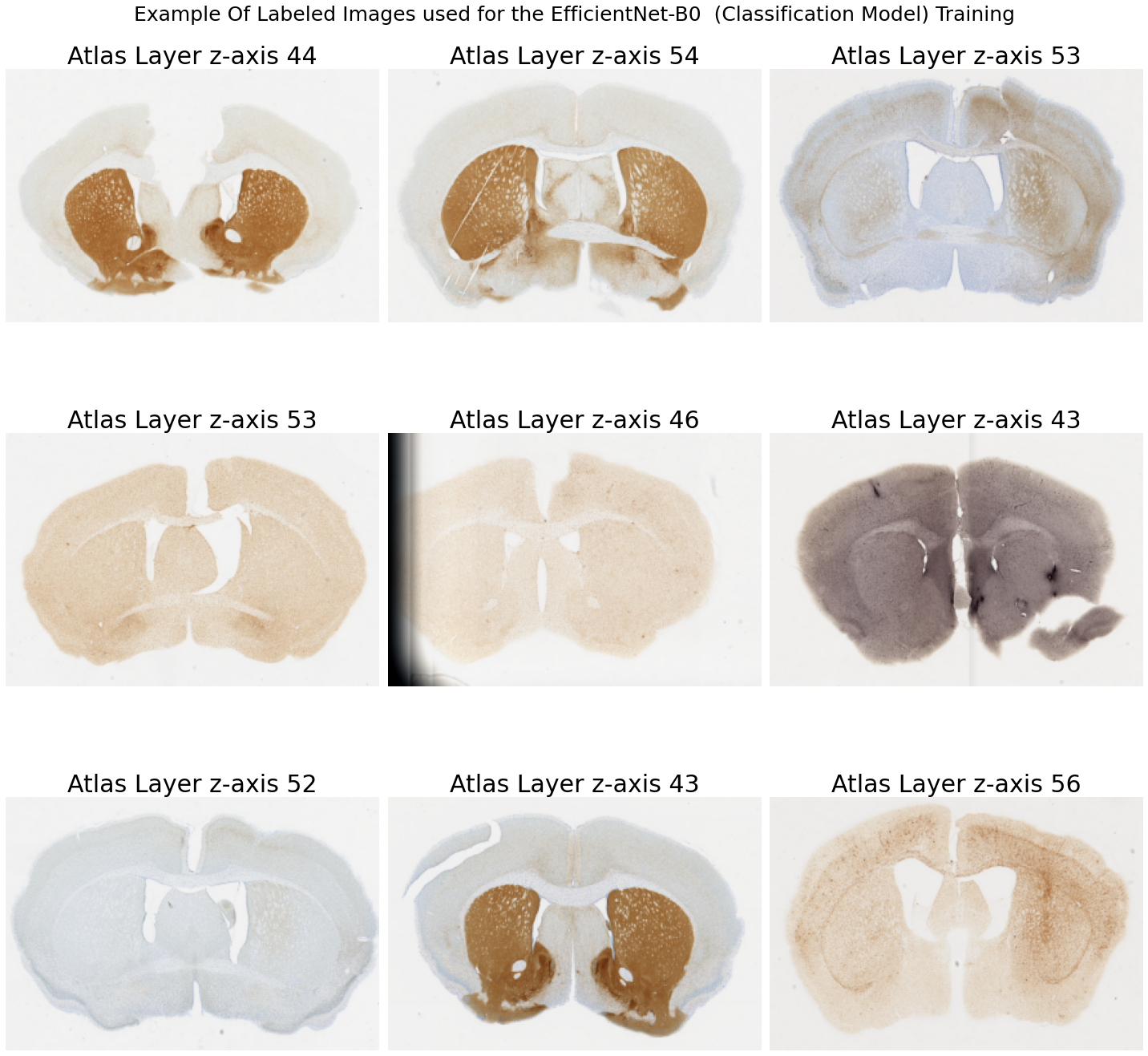


**Figure S4**. Distribution of atlas slices used for training of the brain to layer matching models.


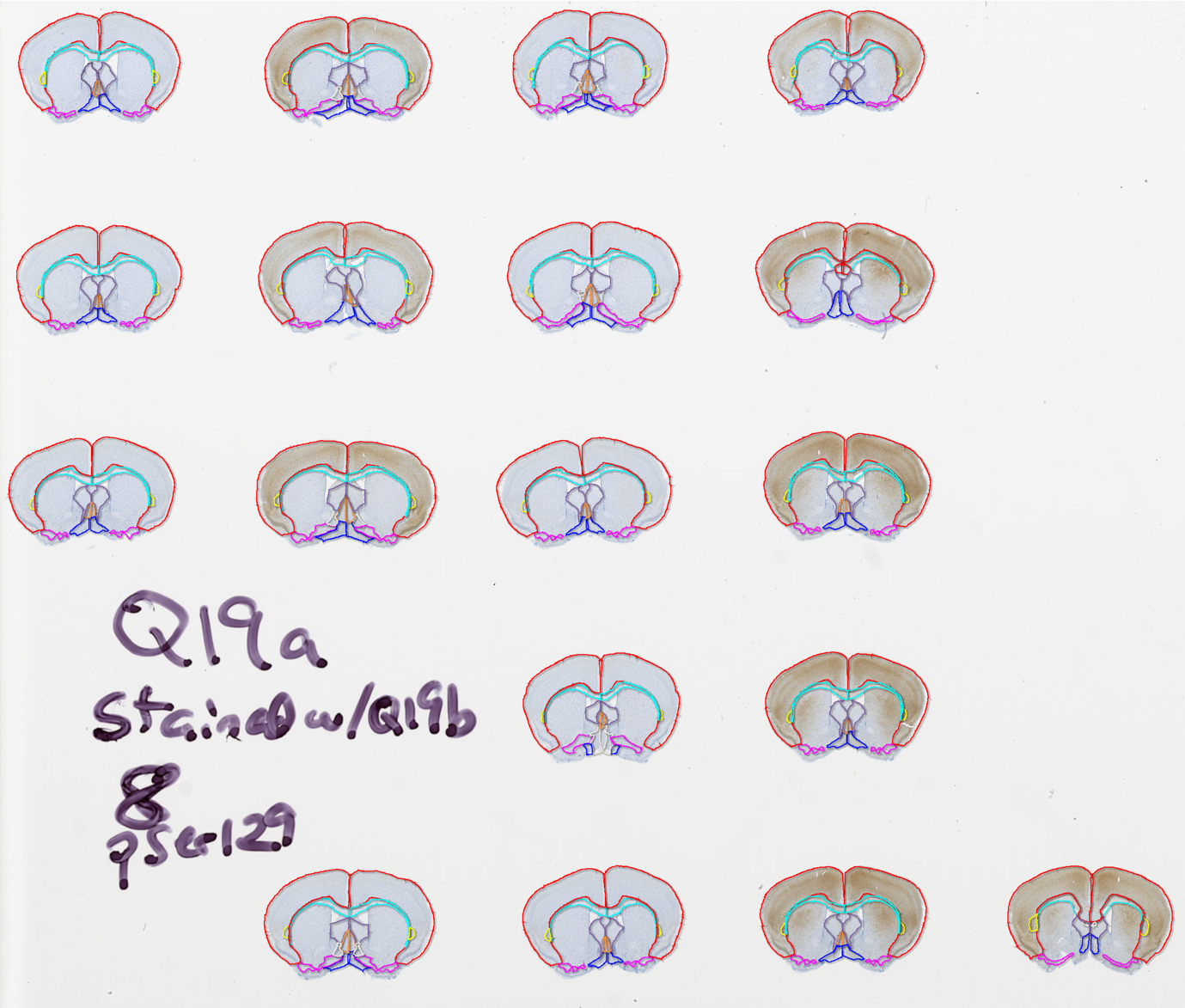


**Figure S5**. Shows the output of AnNoBrainer for a full slide containing 18 brain samples. Each annotated brain region (Corpus Callosum (CC), Cerebral Cortex (CTX), Lateral Septal Nucleus – Rostral Part (LSr), Hypothalamus (HY), Substantia Innominata (SI), Medial Septal Nucleus (MS), Diagonal Band Nucleus (NDB), and Claustrum (CLA)) is depicted in a distinct color to ensure visibility.


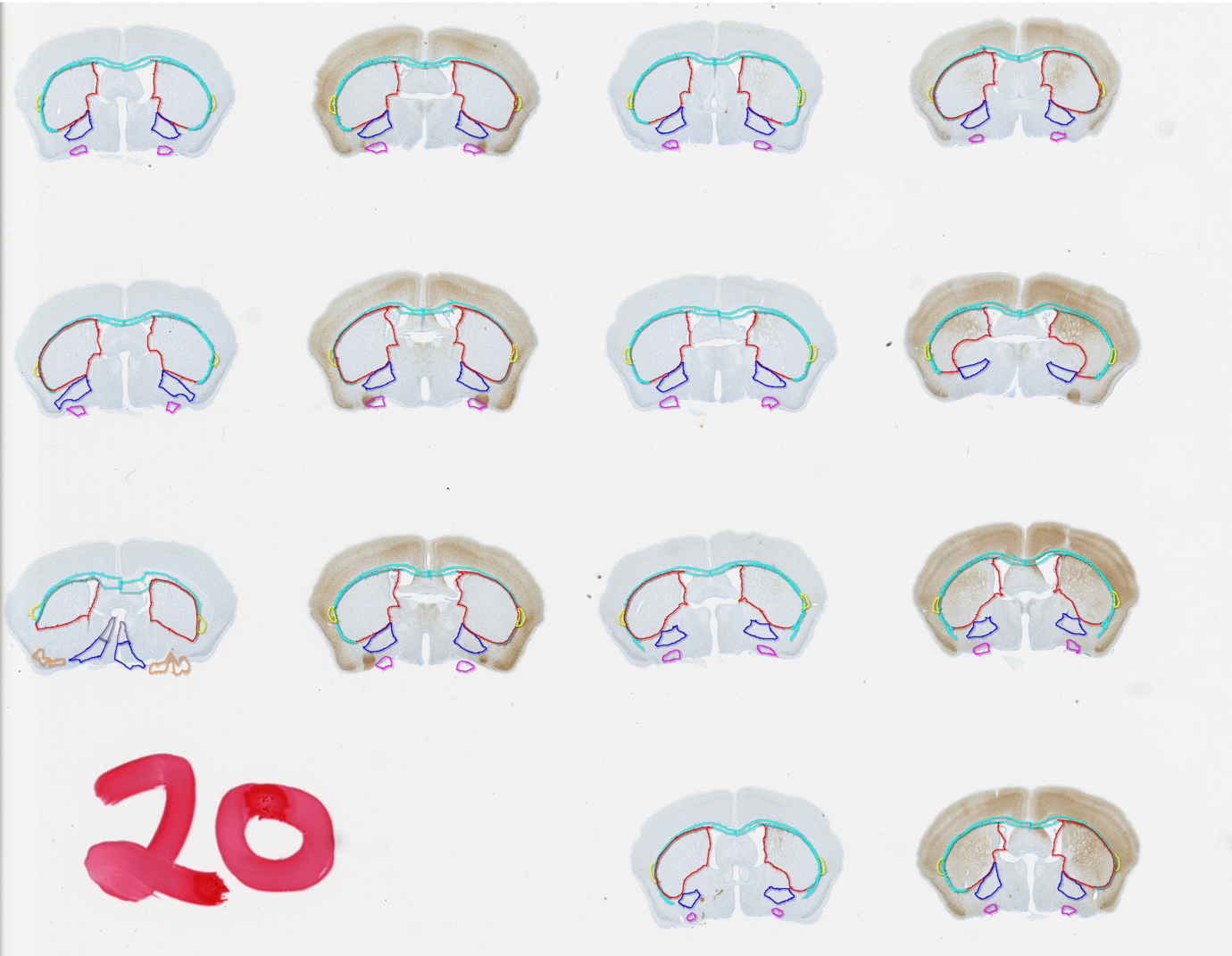


**Figure S6.** Displays the results generated by AnNoBrainer for a complete slide comprising 14 brain samples. Each anatomically annotated brain region (Corpus Callosum (CC), Cerebral Cortex (CTX), Lateral Septal Nucleus – Rostral Part (LSr), Hypothalamus (HY), Substantia Innominata (SI), Medial Septal Nucleus (MS), Diagonal Band Nucleus (NDB), and Claustrum (CLA)) is distinctly colored for clear visualization.
